# Genomic Insights into the Metabolic Traits and Adaptation Mechanisms of Mesophilic *Campylobacteria* Represented by a Novel *Sulfurospirillum* Species from Shallow-Water Hydrothermal Vent

**DOI:** 10.3390/microorganisms14051119

**Published:** 2026-05-14

**Authors:** Xi Du, Mingye Sun, Shan Cheng, Jiang-Shiou Hwang, Rulong Liu, Jiasong Fang, Li Wang

**Affiliations:** 1 College of Oceanography and Ecological Science, Shanghai Ocean University, Shanghai 201316, China; dx476731859@163.com (X.D.); yup_smy99@163.com (M.S.); cwoshiss@163.com (S.C.); rlliu@shou.edu.cn (R.L.); jsfang@shou.edu.cn (J.F.); 2 Institute of Marine Biology, National Taiwan Ocean University, Keelung 202301, Taiwan; jshwang@mail.ntou.edu.tw

**Keywords:** hydrothermal vent, *Campylobacteria*, *Sulfurospirillum*, genomics

## Abstract

Members of the class *Campylobacteria* are microaerophilic bacteria widely distributed across diverse environments and are abundant in hydrothermal systems. However, cultivated representatives, particularly from shallow-water vents, remain limited. Here, we investigated the genomic diversity and environmental adaptation of the genus *Sulfurospirillum*. Phylogenomic analysis revealed a clear separation between terrestrial and marine clades, with relatively few cultured representatives in the marine lineage. Strain 1307, isolated from shallow-water hydrothermal vents, expands the genomic representation of this underexplored clade. Pan-genome analyses based on complete genomes revealed an open pan-genome, indicating ongoing diversification of genus *Sulfurospirillum*. Further comparison between hydrothermal vent (HTV) and non-HTV lineages identified distinct adaptive features. Vent-associated strains are enriched in genes involved in sulfur metabolism, carbon fixation, the glycine cleavage system (GCS), and the biosynthesis of key cofactors (spermidine, thiamine, lipoate, and heme), reflecting metabolic adaptation to hydrothermal environments. Beyond well-established processes such as sulfur metabolism and autotrophic carbon fixation, the widespread presence of the GCS in vent-associated lineages suggests its potential role as an auxiliary carbon fixation pathway under anaerobic conditions. Overall, this study expands the phylogenetic and genomic diversity of *Sulfurospirillum* and offers new insights into the mechanisms underlying environmental adaptation and niche differentiation in vent-associated *Campylobacteria*.

## 1. Introduction

Hydrothermal vents (HTVs) are dynamic systems where seawater circulates through the oceanic crust and is discharged at geologically active sites—such as mid-ocean ridges and plate boundaries—where sufficient heat drives fluid flow [1,2]. As seawater interacts with hot subsurface lithologies, it becomes heated and enriched with reduced inorganic species, including volatiles and metals leached from the rocks [3]. These hydrothermal processes create chemically rich environments that sustain food webs driven by chemolithoautotrophic microorganisms, which derive energy from inorganic compounds [4,5]. HTVs can be broadly classified into two groups based on depth: deep-sea HTVs (>200 m) and shallow-sea hydrothermal vents (SW-HTVs) (<200 m) [5]. Although these two types share similar internal and fluid structures, they differ markedly in biomass, temperature, pH, light availability, and ecological interactions. In SW-HTVs, hydrothermal plumes can reach the surface, influencing surrounding waters and interacting closely with human activities and nearby ecosystems [6]. However, these systems remain relatively understudied. The unique conditions of SW-HTVs—often located along volcanically and tectonically active margins—are shaped by light availability, terrestrial inputs, tidal circulation and atmospheric water inputs, fostering highly diverse and complex microbial communities [1,3].

Members of the class *Campylobacteria* (belonging to phylum *Campylobacterota*, formerly *Epsilonproteobacteria*), are Gram-negative, microaerophilic bacteria widely distributed across diverse environments, including animal hosts, terrestrial habitats, and marine ecosystems such as HTVs. In hydrothermal systems, they are abundant in chimneys, subsurface, and diffuse plumes, and also form symbiotic associations with vent-dwelling metazoans [7]. Based on 16S rRNA gene analyses, *Campylobacteria* can dominate microbial communities in deep-sea HTVs, reaching relative abundances of up to 80~90% in chimney structures [8], as well as in low-temperature overflow zones, subsurface sediments, and plumes [9,10]. They are also highly prevalent in SW-HTVs, particularly in fluids and sediments characterized by moderate temperatures (35–55 °C) and high concentrations of hydrogen sulfide (up to 1.5 mM) and other reduced sulfur compounds [1]. For example, in vents around Kueishan Island, *Campylobacteria* can account for more than 90% of the bacterial community in sediments [11]. Physiological studies of cultured strains from both deep-sea [7,12] and SW-HTVs [13] indicate that they are chemolithoautotrophs with versatile metabolic capabilities, including sulfur oxidation, sulfur/sulfite reduction, reduction of nitrate or nitric oxide to ammonium or nitrogen, as well as hydrogen and formate oxidation.

Despite their ecological importance, relatively few *Campylobacteria* have been obtained in pure culture [14]. Early phylogenetic analyses based on 16S rRNA genes identified several major lineages, including *Nautiliales*, *Arcobacter*, *Sulfurospirillum*, and an environmental sequence branch [7]. Subsequent genome-based classification refined this taxonomy, placing these lineages primarily within the orders *Nautiliales* and *Campylobacterales* [15]. Members of the order *Nautiliales* are typically moderately thermophilic, whereas those of the order *Campylobacterales* are generally adapted to lower temperatures (with the exception of *Hydrogenimonas*) and exhibit greater tolerance to oxygen [16]. Within *Campylobacterales*, genera such as *Sulfurovum* and *Sulfurimonas* have been extensively studied in hydrothermal environments, whereas comparatively fewer studies have focused on genera such as *Sulfurospirillum*. Notably, cultivated representatives of *Sulfurospirillum* from hydrothermal vents remain extremely limited.

Our previous study reported the first *Campylobacteria* isolated from SW-HTVs, extending the known diversity of hydrothermal lineages to include the genus *Sulfurospirillum* [13]. Building on this finding, the present study moves beyond single-strain characterization by establishing a comparative genomic framework to investigate environmental adaptation within the genus. Specifically, we report the genome of a novel species, strain 1307, and perform comprehensive analyses based on complete genomes of cultivated *Sulfurospirillum* strains. By integrating phylogenomics, pan-genome analysis, and functional comparisons, we identify genomic features associated with hydrothermal environments, including unique metabolic and regulatory traits potentially linked to environmental adaptation. Thus, the novelty of this study lies not only in the inclusion of a new genome, but also in the establishment of a comparative framework that reveals environment-associated genomic differentiation within the genus.

## 2. Materials and Methods

### 2.1. Enrichment and Isolation

Strain 1307 was isolated from a sulfur-rich sediment sample collected from the SW-HTVs near Kueishan Island, off Taiwan (121.96232° E, 24.83420° N), and purified using a dilution-to-extinction approach in MMJHS medium [17]. The sampling site was located at a depth of 21 m below sea level, with a temperature of approximately 50 °C and a pH of around 4.7, as previously reported [18]. In subsequent years, sediment samples were collected from the same vent sites for bacterial isolation; however, environmental parameters were not measured. After more than one year of purification, culture purity was confirmed by microscopic examination, and repeated partial sequencing of the 16S rRNA gene using both Sanger and high-throughput sequencing. Strain 1307 has been deposited in the Marine Culture Collection of China (Xiamen, China) under the strain preservation number MCCC 1K08950. Its phylogenetic relationship with affiliated strains has been previously described based on 16S rRNA gene analysis [13].

### 2.2. Genome Sequencing, Assembly and Annotation

Genomic DNA was extracted using the MiniBEST Bacteria Genomic DNA Extraction Kit v3.0 (Takara Bio Inc., Dalian, China) according to the manufacturer’s instructions. DNA concentration was measured using a TBS-380 fluorometer (Turner BioSystems Inc., Sunnyvale, CA, USA). High-quality DNA was sequenced using the PacBio RS II Single Molecule Real Time (SMRT) and Illumina sequencing platforms (Majorbio Bio-pharm Technology Co., Ltd., Shanghai, China). Illumina reads were used to assess the genome quality and correct sequencing errors. After quality trimming, PacBio reads were assembled using NextDenovo [19], which yielded the best assembly quality among the tested assemblers based on metrics including completeness and contiguity. The assembly was subsequently polished using Pilon v1.24 [20] based on Illumina reads. Coding sequences (CDSs) were predicted using Glimmer v3.02 [21], tRNA using tRNA-scan-SE v2.0 [22], and rRNA using Barrnap v0.9 (https://github.com/tseemann/barrnap; accessed on 10 December, 2024). Functional annotation was performed against the Gene Ontology (GO), Clusters of Orthologous Groups (COGs), Kyoto Encyclopedia of Genes and Genomes (KEGG), NR (non-redundant protein sequence database), Swiss-Prot, and Pfam databases. Best hits with an e-value < 10^−5^ were retained for annotation.

### 2.3. Available Genomic Information for Sulfurospirillum

The complete genome sequence of strain 1307 has been deposited in the National Center for Biotechnology Information (NCBI) under accession number CP176011. For phylogenomic analysis, 29 genomes assigned to the genus *Sulfurospirillum* (NCBI Taxonomy ID: 57665) with ≥95% completeness and <5% contamination as assessed by CheckM v1.2.0 [23], including metagenome-assembled genomes (MAGs), were retrieved from the NCBI database (Supporting Materials, Table S1). The genomic phylogenetic trees were constructed using IQ-TREE v3.1.1 [24]. The best-fit substitution model was automatically selected using ModelFinder [25] implemented in IQ-TREE, based on the Bayesian information criterion. The resulting tree was visualized and edited using iTOL v6.9.1 (https://itol.embl.de/; accessed on 2 December, 2024). Taxonomic assignment was further evaluated using GTDB-Tk v2.0.0 [26].

For comparative genomic analyses, only complete genomes were retained to minimize biases associated with incomplete assemblies. A total of 15 complete genomes were included (highlighted in Supplementary Table S1). These include *Sulfurospirillum deleyianum* DSM 6946^T^ [27], *Sulfurospirillum multivorans* DSM 12446^T^ [28], *Sulfurospirillum multivorans* N [29], *Sulfurospirillum barnesii* DSM 10660^T^ [30], *Sulfurospirillum halorespirans* PCE-M2 [31], *Sulfurospirillum cavolei* UCH003 [32], *Sulfurospirillum* sp. UCH001 [32], *Sulfurospirillum* sp. SP [33], *Sulfurospirillum* sp. hDNRA2 [34], *Sulfurospirillum* sp. 1612 [13] and five genomes of *Sulfurospirillum diekertiae* (ACS_TCE_, ACS_DCE_, JPD-1, SL2-2, and SL2-1) [29,35]. All selected genomes are annotated as “complete genome” or “chromosome” in the NCBI database.

### 2.4. Genomic Comparison and Pan-Genome Analysis Among the Complete Genomes

Genome relatedness between strain 1307 and 15 complete genomes of *Sulfurospirillum* was assessed using the average nucleotide identity (ANI), percentage of conserved proteins (POCP), and average amino acid identity (AAI) indices. ANI values were calculated using the pyANI v0.2.12 [36], AAI using CompareM v0.1.2 (http://github.com/dparks1134/CompareM; accessed on 6 April, 2025), and POCP as previously described [37].

Pan-genome analysis was performed using BPGA v1.3 with default settings [38]. Core genes were defined as those shared by all strains, accessory genes as those shared by at least two but not all strains, and unique genes as those present in a single strain. The pan-genome was modeled using a power–law regression of the form (y = A x^b^), where y represents the number of gene clusters and x the number of genomes sequentially added. The exponent b (power–law regression parameter) was used to evaluate pan-genome openness (b < 1 indicates an open pan-genome). Based on the presence/absence of a given accessory and unique gene in “HTV” and “non-HTV” associated environments, Fisher’s exact test was applied to identify genes associated with environment origin using the fisher.test function in R. To account for multiple testing, *p*-values were adjusted using the Benjamin and Hochberg false discovery rate (FDR) method. Furthermore, a binomial distribution model [39] was used to identify significantly enriched COG functions among source-associated genes. An enrichment index, defined as the number of standard deviations from the expected value under binomial distribution, was calculated. An enrichment index > 2 was considered to indicate statistically significant enrichment (*p* < 0.05).

The presence or absence of KEGG modules was also compared across the 16 complete genomes and 15 other additional *Campylobacteria* genomes isolated from deep-sea HTVs. Module completeness was categorized into four levels based on the presence of KEGG orthologs (KOs): absent (no KOs annotated), partially complete (missing ≥ 3 KOs), almost complete (missing 1~2 KOs), and complete (all KOs annotated). These categories were further converted into a numerical scale from 0~4, representing increasing levels of completeness. The resulting module profiles were further analyzed using STAMP v2.1.3 [40].

## 3. Results

### 3.1. Classification Based on Phylogenomic Analysis

**Figure 1:**
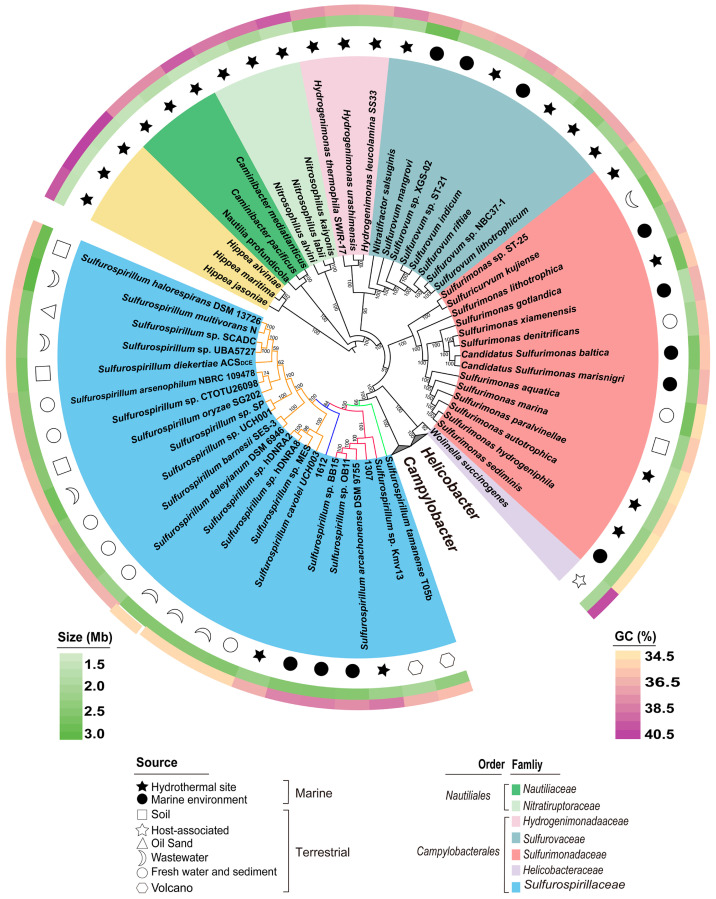
Phylogenomic tree of *Campylobacteria*. A phylogenetic tree was reconstructed using 29 *Sulfurospirillum* genomes with >95% completeness (as assessed by CheckM), together with 60 representative genomes from other *Campylobacteria*. Family-level taxa are indicated by background colors behind genome labels. Branch colors within the family *Sulfurispirillaceae* highlight four subclades. The tree was rooted using three *Hippea* genomes as outgroups. Concentric rings represent, from inner to outer, environmental source categories, GC content (%) and genome size (Mb), respectively. The phylogeny was inferred by maximum likelihood with IQTREE using model LG+F+I+R7, and branch support values estimated from 1000 replicates.

A phylogenomic tree of class *Campylobacteria* genomes was constructed (Figure 1). Notably, members of *Sulfurospirillum* clustered in proximity to host-associated genera such as *Campylobacter*, *Helicobacter,* and *Wolinella*. Within the *Sulfurospirillum* lineage, four well-supported subclades were resolved. One subclade comprised genomes originating from diverse terrestrial environments, including freshwater, soil, sewage, and groundwater (yellow branch in Figure 1). In addition, *Sulfurospirillum* sp. 1612 from SW-HTVs [13] and *Sulfurospirillum tamanense* T05b, an alkaliphilic bacterium isolated from a terrestrial mud volcano [41], each formed independent branches. The remaining genomes, primarily derived from marine environments, clustered into a separate subclade (red branch in Figure 1). These included the newly isolated strains 1307, *Sulfurospirillum arcachonense* from coastal sediment [42], the MAG *Sulfurospirillum* sp. Kmv13 reconstructed from a mud volcano metagenomes [43], and two MAGs (*Sulfurospirillum* sp. OB11 and BB15) recovered from marine bone-degrading microbiomes [44]. In the phylogenomic tree constructed using genomes with 95% completeness (Figure 1) and >90% completeness (Supporting Materials, Figure S1), strain 1307 consistently clustered within a clade lacking cultured representatives, highlighting its importance in expanding our understanding of this lineage within the genus.

**Figure 2:**
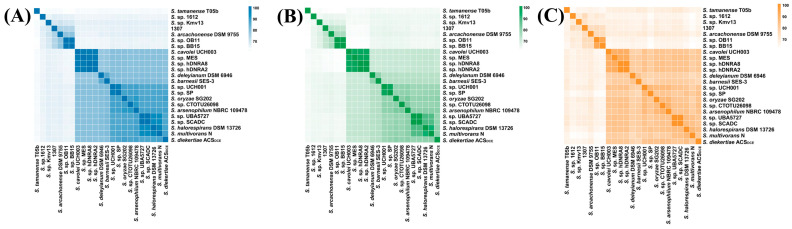
Pairwise genomic similarity among *Sulfurospirillum* genomes. Heatmaps show ANI (**A**) AAI (**B**) and POCP (**C**) values, indicating genomic relatedness among strains.

Genomic similarity analyses based on ANI, AAI and POCP were performed between strain 1307 and all *Sulfurospirillum* genomes shown in Figure 1. Strain 1307 exhibited a maximum ANI of 75.03% with other genomes (Figure 2 & Supporting Materials, Table S2), which is far below the species threshold of 95% ANI [45,46], indicating that it represents a novel species. The AAI value between 1307 and other genomes ranged from 61.70% to 73.21% (Supporting Materials, Table S3), spanning the lower boundary (~65%) generally proposed for genus-level delineation [47]. Notably, AAI values below 65% were primarily observed between strain 1307 and genomes belonging to the terrestrial subclade (yellow branch in Figure 1). The POCP values ranged from 56.32% to 69.98% (Supporting Materials, Table S4), exceeding the commonly used 50% threshold for genus assignment [37]. According to the GTDB taxonymy classification based on genomes, *Sulfurospirillum* sp. 1612 was classified as an unknown genus, but strain 1307 was assigned to the genus *Sulfurospirillum* (Supporting Materials, Table S1). Taken together, these results support the designation of strain 1307 as a novel species within the genus *Sulfurospirillum*. However, its borderline AAI values suggest ambiguity at the genus level. To further resolve this issue, an additional phylogenomic analysis was conducted using *Sulfurospirillum* genomes with ≥90% completeness and <5% contamination (Supporting Materials, Figure S1). The resulting tree revealed a clear phylogenetic separation, with one clade comprising predominantly terrestrial isolates, and another clade—including strain 1307, *Sulfurospirillum* sp. 1612, and MAGs—associated with marine and hydrothermal vent environments. Whether this divergence reflects deep lineage diversification within the genus *Sulfurospirillum* or warrants the establishment of a novel genus remains uncertain and will require additional isolates and genome-resolved analyses.

### 3.2. Genomic Features of the Novel Campylobacteria Isolated from SW-HTVs

The genome of strain 1307 consisted of a single circular chromosome of 2,321,384 base pairs (bps) with an average GC content of 38.40% (Supporting Materials, Figure S2). No plasmids were detected. A total of 2311 open reading frames (ORFs) were predicted, with a combined length of 2,180,871 bps, accounting for approximately 93.95% of the genome. The genome contained 45 tRNA genes and 12 rRNA genes, including four copies each of the 5S rRNA, 16S rRNA and 23S rRNA gene.

Functional annotation was performed based on the COG, KEGG, and NR databases (Supporting Materials, Table S5). A total of 1948 genes (84.29% of the genome) were assigned to COG families spanning 23 functional categories. The most abundant categories were signal transduction mechanisms (Category T; 213 genes), energy production and conversion (Category C; 210 genes), and amino acid transport and metabolism (Category E; 211 genes) (Supporting Materials, Figure S3). KEGG annotation assigned putative functions to 1831 genes (79.23%, Supporting Materials, Table S5), most of which were associated with metabolic pathways (Supporting Materials, Figure S4). Four genomic islands (GIs) were identified in the genome of strain 1307, including GI01 (coordinates 1,733,941-1,746,282, total 12,341 bps), GI02 (1,806,552–1,817,389, total 10,837 bps), GI03 (844,137–849,080, total 4943 bps), and GI04 (coordinates 888,877–902,057, total 13,180 bps) (Supporting Materials, Table S5). These regions are likely derived from horizontal gene transfer. Most genes within these islands encode hypothetical proteins, suggesting the acquisition of novel or uncharacterized functions.

The genome of strain 1307 encodes a diverse set of metabolic pathways involved in sulfur, nitrogen, carbon, and energy metabolism (Supplementary Figure S5 and Table S6). Sulfur metabolism appears to be highly developed. Strain 1307 harbors multiple genes involved in sulfur oxidation, including *sox*, *sqr*, and *fcc*, as well as genes associated with sulfur reduction (*phs and ttr*). Notably, two distinct *sox* gene clusters (*soxABCDXYZ* and *soxCDYZ*) and two *phs* clusters were identified, suggesting functional redundancy and potential flexibility in sulfur transformation pathways (Supporting Materials, Table S6). Nitrogen metabolism pathways indicate the capacity for nitrate reduction and subsequent nitrite conversion to either ammonia or nitrogen, implying versatility in nitrogen utilization under different environmental conditions. Carbon metabolism analysis revealed that strain 1307 possesses most enzymes required for glycolysis but lacks the key enzyme phosphofructokinase (PFK), indicating an incomplete Embden–Meyerhof pathway. In contrast, nearly all genes required for gluconeogenesis are present, enabling the synthesis of fructose-6-phosphate from oxaloacetate. Importantly, the presence of *aclAB* and *oorABCD* genes suggests a functional reductive tricarboxylic acid (rTCA) cycle, supporting the potential for autotrophic CO_2_ fixation. In addition, strain 1307 encodes pathways for the utilization of simple organic substrates, including formate, lactate, and malate, indicating metabolic flexibility between autotrophic and heterotrophic lifestyles. The presence of genes involved in hydrogen metabolism (*hya, hyd, hup*) further suggests the ability to use hydrogen as an electron donor. Energy metabolism is supported by genes encoding hydrogen production (*hyf*) and acetate formation (*ack*-*pta*), indicating potential fermentative capabilities. Furthermore, the presence of cytochrome bd oxidase and the cytochrome bc_1_–bb_3_ supercomplex suggests adaptation to fluctuating oxygen conditions. Finally, strain 1307 encodes only the menaquinone biosynthesis pathway, consistent with its anaerobic or microaerophilic lifestyle. Overall, these genomic features indicate that strain 1307 possesses a versatile metabolic repertoire, with a strong emphasis on sulfur metabolism and the capacity to adapt to dynamic redox conditions characteristic of hydrothermal vent environments.

### 3.3. Pan-Genome Analysis and Functional Traits Associated with Bacterial Adaptation in SW-HTVs

**Figure 3:**
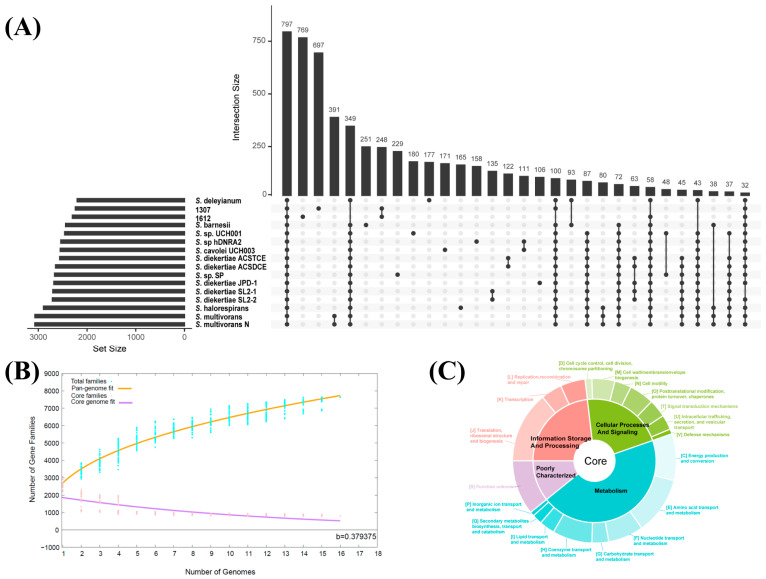
Pan-genome analysis of *Sulfurospirillum*. (**A**) Upset plot showing shared and unique gene clusters across genomes. Black dots represent the presence of gene clusters in specific genome combinations. (**B**) Pan-genome profile trends of the genus *Sulfurospirillum*. (**C**) COG functional annotation of core gene clusters.

A pan-genome analysis was conducted using strain 1307 and 15 publicly available complete genomes of *Sulfurospirillum*. In total, 7632 orthologous gene clusters (GCs) were identified. Among these, 797 core GCs (shared by all genomes) accounted for just over 10% of the pan-genome (Figure 3A). Strain-specific GCs were less abundant, with 769 and 697 unique GCs identified in *Sulfurospirillum* sp. 1612 and strain 1307, respectively. The pan-genome exhibited an open architecture, as indicated by a power–law regression parameter (b = 0.379375), suggesting continuous expansion with the addition of new genomes (Figure 3B). Functional annotation of core genes showed that they were primarily involved in essential cellular processes, such as energy production and conversion, amino acid transport and metabolism, including ribosomal structure and biogenesis (Figure 3C).

**Figure 4:**
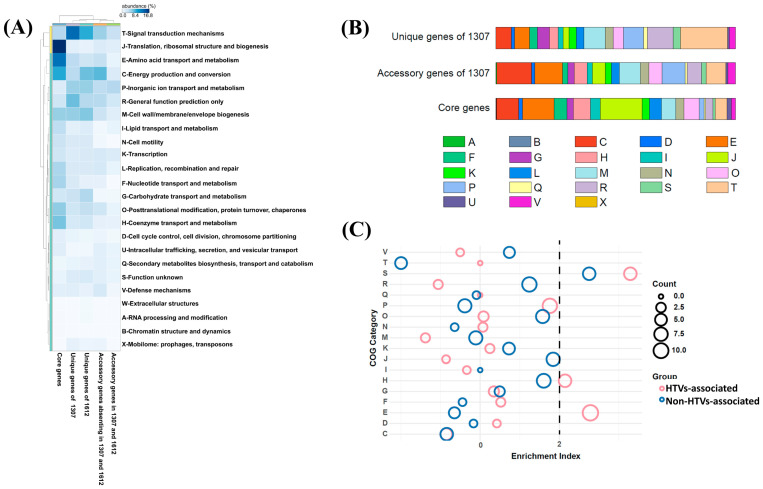
Statistics of the *Sulfurospirillum* pan-genome. (**A**) Distribution of COG level 2 categories for each selected gene set. (**B**) Distribution of COG functional categories among the core, accessory and unique genes of strain 1307. (**C**) Enrichment analysis of COG functional categories in genomes from hydrothermal vent (HTV) versus non-HTV environmental sources. An enrichment index greater than 2 (black dashed line) indicates significant enrichment of a given COG category. The size of each circle corresponds to the logarithm of the number of genes annotated to the respective COG category. Abbreviations of COG categories are described below. A: RNA processing and modification; C: Energy production and conversion; D: Cell cycle control, cell division, chromosome partitioning; E: Amino acid transport and metabolism; F: Nucleotide transport and metabolism; G: Carbohydrate transport and metabolism; H: Coenzyme transport and metabolism; I: Lipid transport and metabolism; J: Translation, ribosomal structure, and biogenesis; K: Transcription; L: Replication, recombination, and repair; M: Cell wall/membrane/envelope biogenesis; N: Cell motility; O: Posttranslational modification, protein turnover, chaperones; P: Inorganic ion transport and metabolism; Q: Secondary metabolites biosynthesis, transport, and catabolism; S: Function unknown; T: Signal transduction mechanisms; U: Intracellular trafficking, secretion, and vesicular transport; V: Defense mechanisms; W: Extracellular structures.

Among the 697 strain-specific GCs in strain 1307, 54.95% were annotated in KEGG and 61.69% were assigned to COG categories (Supporting Materials, Table S7). Genes related to signal transduction (Category T) were notably higher in the unique gene set compared to accessory and core genes (Figure 4A,B). These genes include diverse regulatory systems such as the histidine kinase involved in chemotaxis and quorum sensing (COG0642, COG3920, COG5002, COG2972), cyclic-di-GMP signaling components (COG5001, COG2199, COG2200, COG3437, COG2206, COG3434), chemotaxis receptors (COG0840), stress response regulators (COG0589, COG1366, COG2208, COG1776), and the toxin–antitoxin system (COG2337), indicating enhanced regulatory capacity (Supporting Materials, Table S8). In addition, genes involved in inorganic ion transport and metabolism (Category P) were more prevalent in both the unique and accessory gene sets than in the core genes. These included ABC-type transport systems for dipeptide, phosphonate, cobalt and other ions (COG1132, COG3842, COG0444, COG0601, COG3221, COG3639, COG4107, COG3638, COG0310), ion channels (COG1283, COG3004, COG1226, COG0598), phosphate/phosphonate metabolic enzymes (COG3454, COG3626, COG4778, COG3625, COG3709, COG3624), sulfotransferases (COG2897, COG0607), nitrite reductase cytochrome subunit (COG3303), and metal chaperones (COG2608, COG4097) (Supporting Materials, Table S8). These features suggest that strain 1307 may possesses specialized regulatory, and metabolic capabilities that may facilitate adaptation to hydrothermal environments. Beyond Categories T and P, comparative functional profiling revealed that unique genes of strain 1307 were also enriched in Category M (cell wall/membrane/envelope biogenesis) (Figure 4A and Supporting Materials, Table S9). Notably, genes involved in energy production and conversion (Category C) were most abundant in the accessory gene set shared by hydrothermal strains 1307 and *Sulfurospirillum* sp. 1612, suggesting enhanced metabolic flexibility and energy utilization strategies adapted to hydrothermal environments (Figure 4).

To investigate environmental adaptation, Fisher’s exact tests were performed on the distribution of accessory and unique GCs. Based on isolation sources, strains were grouped into HTV-associated (strains 1307 and *Sulfurospirillum* sp. 1612) and non-HTV-associated groups. A total of 139 GCs showed significant associations with environmental origin (*p* < 0.05), including 57 GCs specific to HTV-associated strains and 82 GCs specific to non-HTV strains (Supporting Materials, Table S10). Genes enriched in HTV-associated strains were primarily involved in amino acid metabolism (Category E) and coenzyme metabolism (Category H), including glycine cleavage system (COG1003, COG0403, COG0404, COG0509), polyamine transport and amino acid metabolism-related genes (COG0687, COG1177, COG1176, COG0010, COG2008, COG2423), and lipoate (COG0320, COG0095) and thiamine biosynthesis (COG0301, COG4143, COG3840). In contrast, genes assigned to the category of unknown function (Category S) were predominantly in non-hydrothermal strains (Figure 4C).

### 3.4. Comparative Analysis of KEGG Modules (KMs)

**Figure 5:**
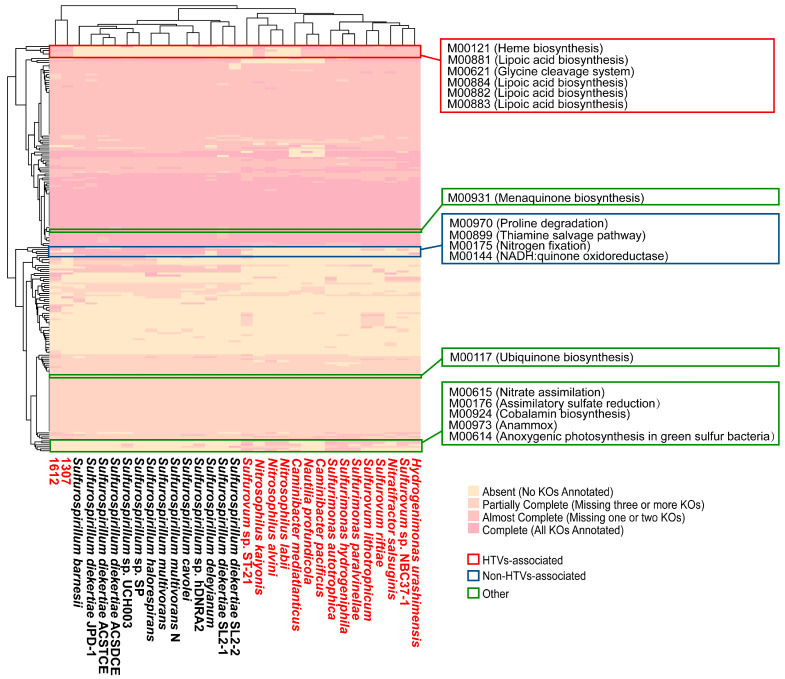
KEGG module profiles in *Campylobacteria*. Columns represent genomes and rows correspond to KEGG modules. Hydrothermal vents strains are highlighted in red. Module completeness is classified as absent (no KOs annotated), partially complete (missing ≥ 3 KOs), almost complete (missing 1~2 KOs), or complete (all KOs annotated) based on KO coverage. Modules associated with hydrothermal vents environments are highlighted with red box. Details module composition is provided in Supporting Material, Figure S6.

A total of 204 KEGG modules (KMs) were identified across 16 *Sulfurospirillum* complete genomes and 15 additional *Campylobacteria* genomes from deep-sea HTVs (Supporting Materials, Table S11). Based on their distribution patterns, hierarchical clustering revealed that SW-HTV-associated strains, including *Sulfurospirillum* sp. 1612 and strain 1307, formed a distinct cluster (top dendrogram in Figure 5). In contrast, the remaining genomes were separated into two major clusters: one comprising non-HTV *Sulfurospirillum* strains and the other consisting of deep-sea HTV-associated *Campylobacteria*. Module-based functional profiling revealed clear environmental differentiation among lineages. Modules involved in heme biosynthesis (M00121), glycine cleavage systems (M00621), and lipoic acid biosynthesis (M00881~M00884, mainly represented by *lipA* [K03644]), were significantly enriched in HTV-associated strains (red box in Figure 5 and Supporting Materials, Table S12). In contrast, non-HTV *Sulfurospirillum* genomes were enriched in pathways such as proline degradation (M00970), thiamine salvage (M00899), nitrogen fixation (M00175) and oxidoreductase activity (M00144) (blue box in Figure 5 & Supporting Materials, Table S12). Furthermore, partial proteins of the heme biosynthesis (M00847), nitrate assimilation (M00615), assimilatory sulfate reduction (M00176), cobalamin biosynthesis (M00924), anammox (M00973), and anoxygenic photosynthesis (M00614) were frequently detected in deep-sea HTV-associated *Campylobacteria* but were absent in the genus *Sulfurospirillum* (green box in Figure 5), highlighting lineage-specific adaptations.

### 3.5. Respiratory and Metabolic Versatility of Sulfurospirillum

**Figure 6:**
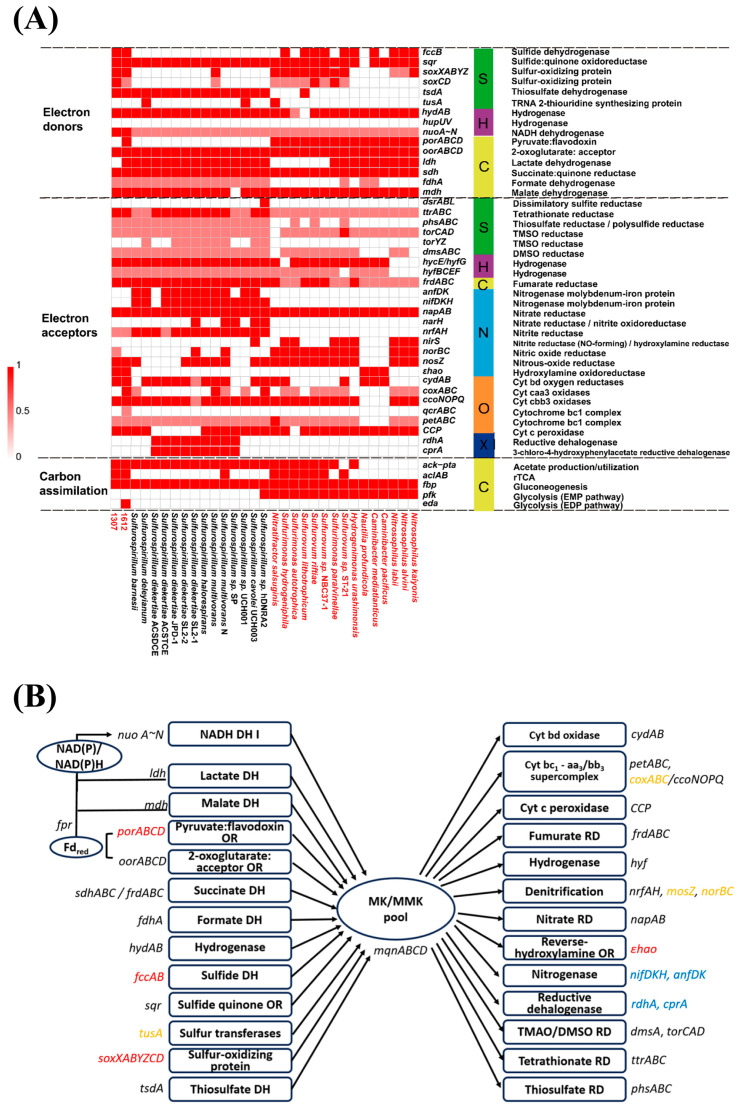
Major functional genes involved in energy conversation in *Sulfurospirillum*. (**A**) Distribution of genes involved in carbon, sulfur, and nitrogen cycles across genomes. Hydrothermal vent strains are indicated in red. Gene completeness is categorized as complete, partial, or absent. (**B**) Schematic of the electron transport chain. Black arrows indicate electron flow. Gene distribution is color-coded as follows: blue, unique to non-HTV strains; red, specific to HTV strains 1307 and *Sulfurospirillum* sp. 1612; orange, present in a subset of strains; black, conserved across all genomes. Abbreviations: MK, menaquinone; MMK, monomethylmenaquinone; Fd, ferrodoxin or flavodoxin; cyt c, cytochrome c; DH: dehydrogenase; OR: oxidoreductase; RD: reducatase.

Genomic analysis revealed substantial metabolic flexibility within the genus *Sulfurospirillum*. Across HTVs-associated strains, including both shallow- and deep-sea isolates, several conserved metabolic features were identified (Figure 6A). These strains consistently harbor genes involved in carbon assimilation (*aclAB*), sulfur oxidation (*sqr*, *fccB*, *soxABCDYZ*), nitrate reduction (*nap*), and partial denitrification (*nos* genes, involving the reduction of N_2_O to N_2_). Notably, the potential for CO_2_ fixation via the rTCA cycle, indicated by the presence of *aclAB* was restricted to *S. multivorans* and the HTVs-associated strains 1307 and *Sulfurospirillum* sp. 1612 (Figure 6A), suggesting a link between autotrophic capacity and specific ecological niches. Although strain 1307 was isolated using MMJHS medium with CO_2_ or sodium carbonate as the sole carbon source, long-term subculturing (approximately two years with transfers every three months) indicated a clear physiological response to different electron donors. Under conditions with elemental sulfur as the sole electron acceptor, strain 1307 showed no obvious response to H_2_, whereas a rapid growth response was observed in the presence of formate (Supporting Materials, Figure S7), suggesting a preferential utilization of formate as an electron donor under the tested conditions.

Comparative analysis between HTVs-associated and non-HTVs *Sulfurospirillum* strains revealed clear metabolic differentiation. Most non-HTVs *Sulfurospirillum* strains possessed complete *nrfAH* genes required for ammonification (Figure 6A). In contrast, strain 1307 and *Sulfurospirillum* sp. 1612 only encoded *nrfH*, lacking the catalytic subunit *nrfA*. Instead, these strains may rely on the *Epsilonproteobacterial* hydroxylamine oxidoreductase (*ε-hao*) to perform ammonification via an alternative ammonification pathway, reducing nitrite to hydroxylamine. Although *Sulfurospirillum* sp. 1612 and strain 1307 lacked *nrfA* (KEGG-defined NrfAH system), COG3303 was detected in strain 1307 based on COG annotation. Phylogenetic analysis of COG3303, *nrfA*, and ε-hao homologs may further clarify their evolutionary relationships and potential functional divergence in nitrite reduction pathways. Furthermore, hydrothermal strains encoded additional sulfur metabolism genes, such as *sox* and *fcc*, indicating an expanded capacity to utilize diverse reduced sulfur compounds. In contrast, non-HTVs strains exhibited a greater prevalence of nitrogen fixation and organohalide respiration-related traits (as described above), reflecting adaptation to terrestrial or contaminated environments.

At the genus level, members of *Sulfurospirillum* share a conserved yet versatile respiratory framework (Figure 6B). Most species are chemoorganotrophs capable of oxidizing substrates such as succinate, fumarate, malate, lactate, formate, hydrogen, and reduced sulfur compounds, supported by electron donor-associated genes (*mdh*, *sdh*, *fdh*, *hyd*, *sqr*, *tsd*). Their genomes encode a wide array of terminal oxidases (cydAB, petABC, *ccoNOPQ*, CCP) and reductases, including fumarate reductase (*frdABC*), periplasmic nitrate reductase (*napAB*), and multiple sulfur respiration enzymes (*dmsA*, *torCAD*, *ttrABC*, *phsABC*). All genomes contained the proton-pumping NADH dehydrogenase (NDH-1, encoded by *nuoABCDEFGHIJKLMN*), while alternative NADH dehydrogenases (*ndh* and *nqr*) were absent. These electron transfer systems form flexible branched respiratory chains, enabling coupling between diverse dehydrogenases and terminal reductases or oxidases depending on environmental conditions. The absence of low affinity oxygen reductases (caa_3_-type *coxABC*) in 1307 and several *Sulfurospirillum* suggested a reduced capacity to adapt to high oxygen concentration compared to *Sulfurospirillum* sp. 1612, which retains this high affinity oxidase system.

## 4. Discussion

*Sulfurospirillum* exhibits considerable genomic diversity that correlates with environmental origin. It is globally distributed and commonly found in sediments, groundwater, and soils, particularly in environments contaminated with organohalides, arsenate, or selenate, or enriched in sulfur compounds [48]. Phylogenomic analysis revealed that two major lineages: a marine lineage with higher GC content and a terrestrial lineage with lower GC content (Figure 1), consistent with previous 16S rDNA based classification. Within this framework, earlier studies resolved three clades, including a marine clade (*S. arcachonense*, *Sulfurospirillum carboxydovorans*, and *Sulfurospirillum* sp. AM-N), the terrestrial clade (including *S. multivorans*) and a third clade with *Sulfurospirillum alkalitolerans* [48]. Together with *Sulfurospirillum* sp. 1612, previously isolated from the SW-HTVs [13], the characterization of strain 1307 expands the known diversity of mesophilic order Campylobacterales in hydrothermal environments and increases the number of HTV-associated *Sulfurospirillum* genomes to two. Compared with *Sulfurospirillum* sp. 1612, strain 1307 possesses a smaller genome size (2,321,384 bp vs. 2,377,931 bp), a higher G+C content (38.40% vs. 36.50%) and fewer coding sequences (2311 genes vs. 2365 genes). The average gene lengths were similar between the two strains: 944 bp for strain 1307 and 942 bp for *Sulfurospirillum* sp. 1612, indicating comparable coding density. Notably, strain 1307 contains a greater number of rRNAs, tRNAs and small RNA genes than *Sulfurospirillum* sp. 1612 (Supporting Materials, Table S5). With the increasing availability of genomes from cultivated representatives, genus *Sulfurospirillum* provides a valuable model for pan-genomic and evolutionary analyses.

Our comparative genomics further highlights specialized adaptation of *Campylobacteria* strains to hydrothermal vent environments. As chemolithoautotrophic bacteria play key roles in HTVs [49], energy metabolism provides a central framework for understanding their environmental adaptation. Within the genus *Sulfurospirillum*, only HTVs-associated strains encode genes for autotrophic carbon fixation. Strain 1307 and *Sulfurospirillum* sp. 1612 both harbor key genes of the rTCA cycle (*aclAB*), suggesting their potential for chemolithoautotrophic carbon fixation. In agreement with these genomic predictions, physiological experiments confirmed that *Sulfurospirillum* sp. 1612 is capable of growth with CO_2_ as the sole carbon source [13], whereas most other *Sulfurospirillum* species are chemolithoheterotrophic [48]. Consistently, these vent-associated genomes also exhibit an expanded repertoire of sulfur metabolism genes, including *sox* and *fcc*, indicating adaptation to sulfur-rich vent conditions. Various sulfur compounds, such as sulfide and elemental sulfur (S^0^), are widely distributed in hydrothermal systems [7,49]. Sulfur isotope studies have demonstrated that microbial-mediated sulfur transformation processes are highly active in hydrothermal systems [50]. Accordingly, dissimilatory sulfur metabolism has been recognized as a key biogeochemical process in these environments [51]. Together, these results indicate that hydrothermal vent lineages within the genus may have evolved enhanced metabolic flexibility toward autotrophic lifestyles.

Notably, vent-originating strains exhibited a significant enrichment of genes associated with the glycine cleavage system (GCS). Also known as the glycine decarboxylase complex, the GCS catalyzes the reversible cleavage of glycine to CO_2_, methylene-tetrahydrofolate and ammonia [52]. In anaerobic microorganisms, this system can operate bidirectionally, either functioning as an electron sink in the reductive carboxylation direction to support autotrophic growth [53], or oxidizing glycine to generate NAD(P)H for cellular metabolism, as observed in the photoheterotrophic bacterium *Chloroflexus aurantiacus* under H_2_ limited conditions [54]. Glycine, as the simplest amino acid, can be abiotically synthesized under hydrothermal conditions and is commonly enriched in hydrothermal fluids. Laboratory and field studies have demonstrated its efficient formation and elevated concentrations in systems such as the Mariana Trough and Red Sea brines [55,56,57,58,59,60,61], suggesting that it represents an environmentally relevant substrate in these ecosystems. Consistently, the GCS module (M00621) was widely detected in *Campylobacteria* from hydrothermal vents (Figure 5) and was significantly enriched in the hydrothermal-associated group of the *Sulfurospirillum* pangenome (Supporting Materials, Table S11). However, whether the GCS in strain 1307 primarily functions in CO_2_ fixation or glycine oxidation remains unclear, and further experiments (e.g., ^13^C tracing) will be required to resolve its physiological role. Beyond the GCS, hydrothermal strains also showed enrichment of genes involved in spermidine, thiamine, lipoate, and heme biosynthesis compared with other *Sulfurospirillum* genomes (Figure 5; Supporting Materials, Tables S10 and S12), suggesting additional metabolic adaptations to extreme environments. Together, these findings point to a coordinated reconfiguration of carbon metabolism and cofactor biosynthesis in vent-associated lineages, although their specific contributions to energy conservation and ecological fitness remain to be elucidated.

The genomic features of *Sulfurospirillum* sp. highlight their adaptive potential to microoxic and anoxic environments. Nonpathogenic *Campylobacteria* are typically associated with aphotic habitats and are enriched in redoxclines, which represent interface between oxic and anoxic zones [8]. Consistent with this ecological distribution, *Sulfurospirillum* species are capable of utilizing a wide range of terminal electron acceptors in addition to O_2_. A key feature underlying this adaptation is their quinone composition. Similar to most *Campylobacteria* [62], *Sulfurospirillum* predominantly produces menaquinone (MK) and its methylated derivative, monomethylmenaquinone (MMK). Compared with ubiquinone (UQ), MK has a lower redox midpoint potential, making it more suitable for anaerobic respiration [63]. For example, although *Escherichia coli* can synthesize both UQ and MK, it preferentially utilizes UQ during aerobic respiration [64], while MK and demethylmenaquinone (DMK) dominate under anaerobic conditions [65]. In our dataset, all *Sulfurospirillum* genomes encoded a complete modified futalosine pathway for MK biosynthesis (KEGG M00931, *mqnABCD*), whereas the ubiquinone biosynthesis pathway (M00117) was incomplete (Figure 5 & Supporting Materials, Table S11). Consistently, physiological assays of *Sulfurospirillum* sp. 1612 revealed that its quinone pool is dominated by MMK6 (95.11%), with a minor proportion of MK6 (4.89%) when grown with elemental sulfur or nitrate as terminal electron acceptors [13]. MMK is relatively rare among prokaryotes and has been reported in only a limited number of bacterial and archaeal groups [66]. Methylation of MK to MMK further lowers its redox potential [67], enabling respiration with molecules with a low redox potential such as polysulfide or sulfite. In the model MMK-producing bacterium *Wolinella succinogenes* (belonging to class *Campylobacteria*), polysulfide respiration depends on MMK6, which interacts with the membrane anchor PsrC of the polysulfide reductase complex (PsrABC) [68]. In addition to quinone composition, terminal oxidases further support adaptation to microoxic conditions. Most *Sulfurospirillum* genomes encode three three distinct oxidases: the cyt bc_1_-bb_3_ supercomplex, the cyt bd oxidase and the cyt c peroxidase (Figure 6B). Heme-copper oxidase (e.g., aa_3_/bb_3_-type) receive electrons from cytochrome c via the quinone-interacting cyt bc_1_ complex, whereas cyt bd oxidase directly accepts electrons from the quinol pool and lacks copper centers. Notably, the high-affinity cbb_3_-type oxidase and the cyt bd oxidase [69], together with cyt c peroxidase—which detoxifies partially reduced toxic H_2_O_2_ to water—collectively enable efficient respiration and oxidative stress tolerance under low-oxygen conditions.

## 5. Conclusions

In this study, we performed a comprehensive genomic and comparative analysis of *Sulfurospirillum* sp. 1307 isolated from SW-HTVs. Genome-based taxonomic metrics consistently support its designation as a novel species, with ANI values well below the species threshold, while AAI and POCP place it near the lower boundary of the genus *Sulfurospirillum*, suggesting a phylogenetically distinct and boundary-positioned lineage. Comparative genomics revealed that strain 1307 possesses abundant strain-specific genes, with a notable enrichment in signal transduction functions. Furthermore, HTVs-associated strains showed consistent enrichment of genes involved in sulfur metabolism, carbon fixation, the glycine cleavage system, and the biosynthesis of key cofactors such as spermidine, thiamine, lipoate, and heme biosynthesis, indicating enhanced metabolic flexibility and adaptation to vent environments. These features underscore the ecological significance of *Sulfurospirillum* in hydrothermal systems, particularly in mediating carbon, nitrogen, and sulfur cycling. Notably, the borderline AAI values observed between strain 1307 and other members of the genus indicate potential taxonomic ambiguity at the genus level, suggesting that it may represent an early-diverging lineage. Resolving whether this divergence reflects deep intra-genus diversification or warrants the establishment of a novel genus will require additional isolates and genome-resolved analyses. Overall, this study provides new insights into the genomic basis of environmental adaptation mechanisms and niche differentiation within *Sulfurospirillum* and establishes a foundation for future genome-resolved phylogenomic and functional investigations.

## Data Availability

The assembled circular genomic data of strain 1307 has been deposited in the NCBI (National Center for Biotechnology Information) under accession number CP176011.

## Supplementary Materials

The following supporting information can be downloaded at: https://www.mdpi.com/article/10.3390/microorganisms14051119/s1, Figure S1: Phylogenomic tree of *Campylobacteria* based on *Sulfurospirillum* genomes with >90% completeness; Figure S2: Graphical circle map of the 1307 chromosome; Figure S3: COG functional classification of strain 1307 genome; Figure S4: KEGG annotation of the genome of strain 1307; Figure S5: Overview of central metabolism pathways in strain 1307; Figure S6: Comparison of KEGG modules between HTVs and non-HTVs strains; Figure S7: The growth curves for the Electron donor testing; Table S1: List of *Campylobacteria* strains used for phylogenetic tree construction; Table S2 The ANI values between our isolates and closely related *Sulfurospirillum* genomes; Table S3 The AAI values between our isolates and closely related *Sulfurospirillum* genomes; Table S4 The POCP values between our isolates and closely related *Sulfurospirillum* genomes; Table S5 Genome features of strain 1307 and *Sulfurospirillum* sp. 1612; Table S6 Detailed analysis of genes involved in carbon, nitrogen, sulfur, and oxygen metabolism in strain 1307; Table S7 Distribution of core, accessory, and unique genes in the pan-genome; Table S8 COG categories significantly enriched in “Unique genes of 1307” compared to “Core genes”; Table S9 Differences in the number of unique gene, accessory gene and core genes in COGs; Table S10 The GCs with significant differences between HTVs strains and non-HTVs strains; Table S11 KEGG module profiles for 16 complete *Sulfurospirillum* genomes and 15 additional *Campylobacteria* genomes isolated from deep-sea hydrothermal vents; Table S12 The KEGG modules with significant differences between HTVs strains and non-HTVs strains.

## Abbreviations

The following abbreviations are used in this manuscript:

HTVs	Hydrothermal vents
SW-HTVs	Shallow-sea hydrothermal vents
MAGs	Metagenome-assembled genomes
MK	Menaquinone
MMK	Monomethylmenaquinone
UQ	Ubiquinone
