# Supplementary material for: Genomic Insights into the Metabolic Traits and Adaptation Mechanisms of Mesophilic Campylobacteria Represented by a Novel Sulfurospirillum Species from Shallow-Water Hydrothermal Vent
Source: Microorganisms. 2026 May 14;14(5):1119. doi: 10.3390/microorganisms14051119 (PMC13209400; doi:10.3390/microorganisms14051119)
Supplement: Supplementary file 1 [file microorganisms-14-01119-s001.zip › Supporting Figures.pdf]

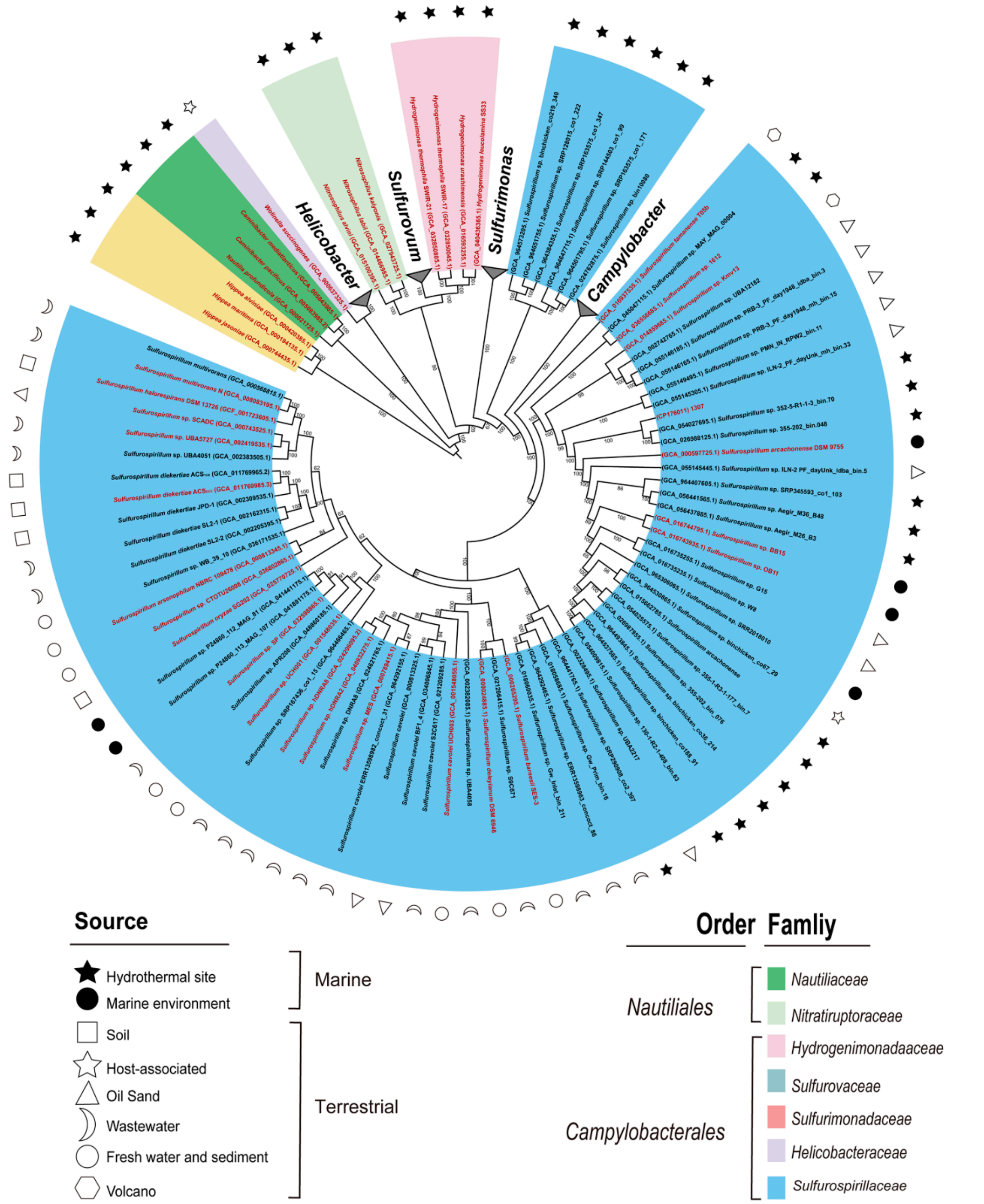

Figure S1 Phylogenomic tree of Campylobacteria based on *Sulfurospirillum* genomes with >90% completeness. A phylogenetic tree was reconstructed using 74 *Sulfurospirillum* genomes with >90% completeness and <5% contamination (as assessed by CheckM), together with representative complete genomes from other Campylobacteria. Family-level taxa are indicated by background colors behind genome labels. The tree was rooted using three genomes from the genus *Hippea* as outgroups. Genomes highlighted in red correspond to those shown in Fig. 1. The phylogeny was inferred using maximum likelihood in IQ-TREE under the LG+F+I+R7 model, and branch support values were estimated from 1,000 bootstrap replicates.

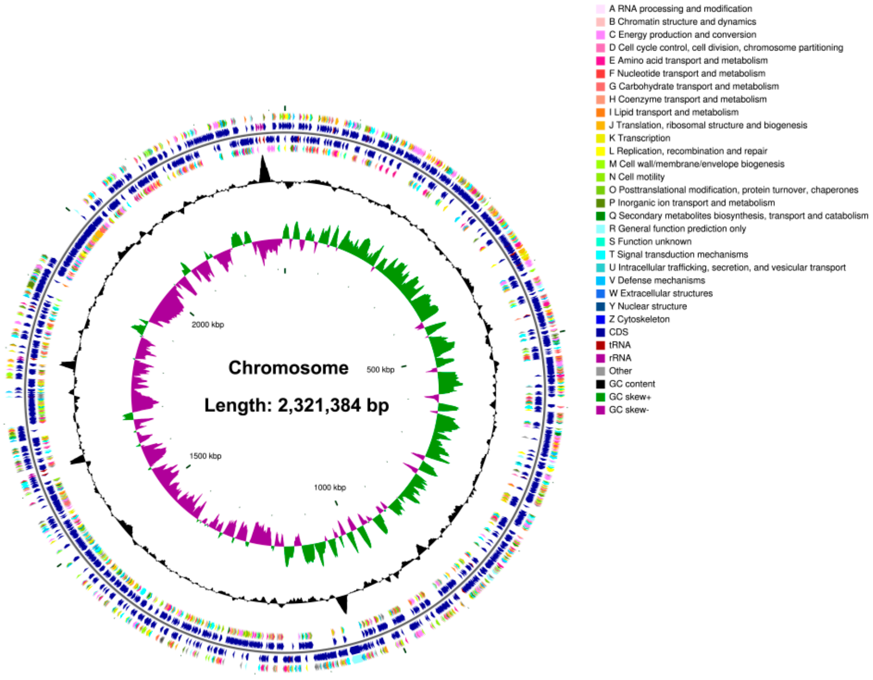

Figure S2 Graphical circle map of the 1307 chromosome. From the inside to the outside, the first circle represents the scale; the second circle represents the GC skew; the third circle represents the GC content; the fourth and seventh circles represent the COG annotation categories with different colours; the fifth and sixth circles represent the position of CDs, tRNA, and rRNA in the genome.

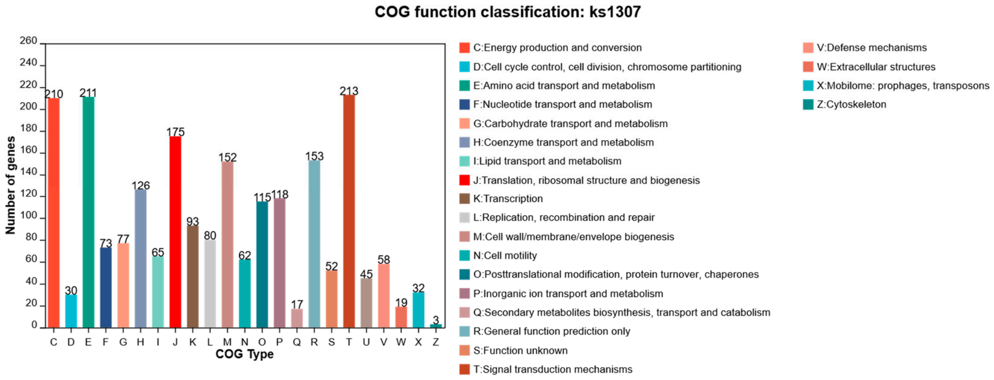

Figure S3 COG functional classification of strain 1307 genome. The x-axis represents COG categories, and the y-axis indicates the number of genes in each category. Functional descriptions of COG categories are provided in the right panel.

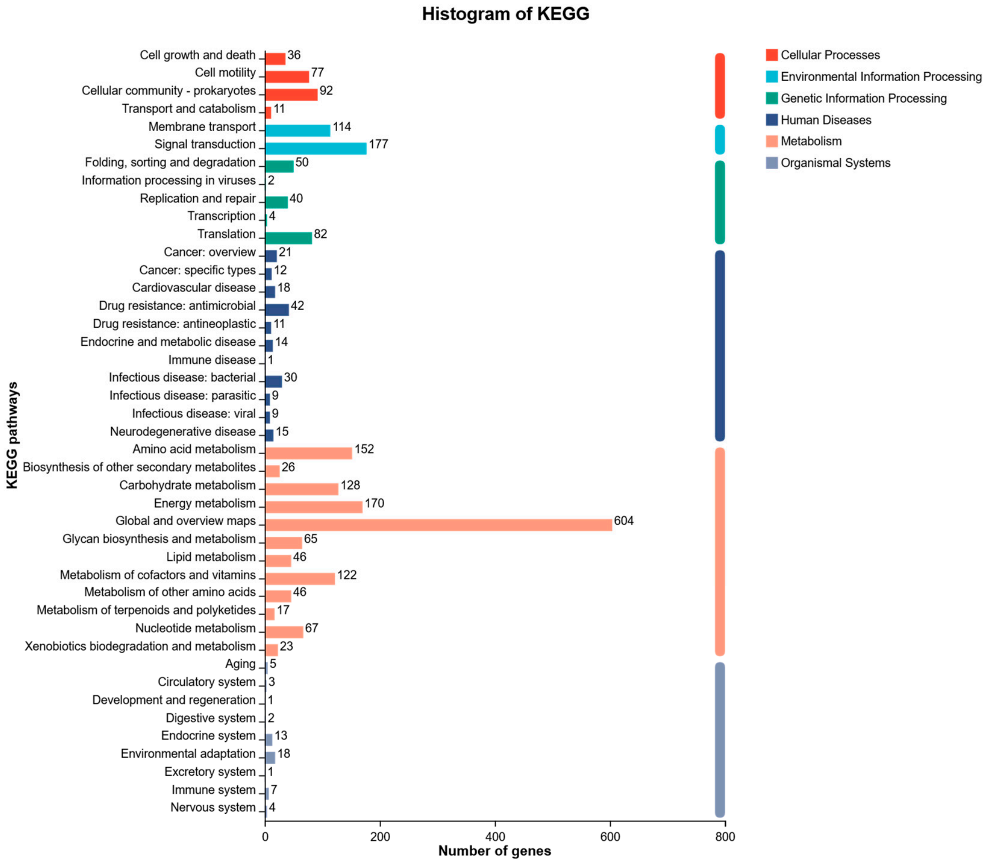

Figure S4. KEGG annotation of the genome of strain 1307. Level 2 KEGG pathways are shown on the y-axis, with bar colors indicating Level 1 functional categories.

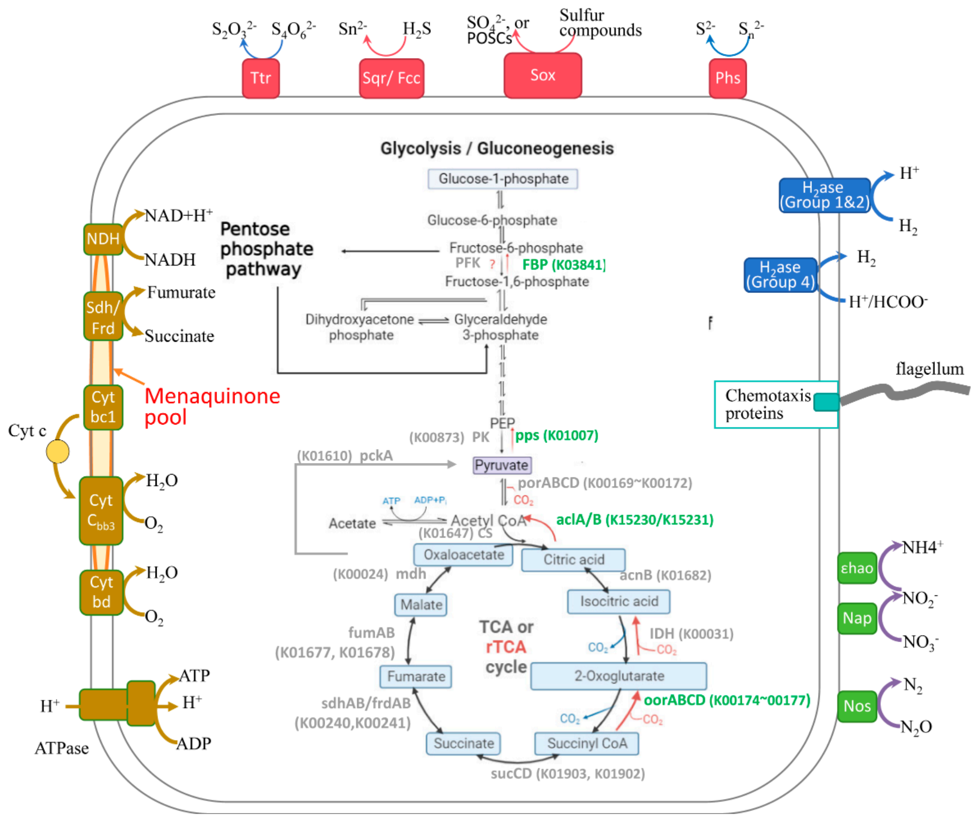

Figure S5 Overview of central metabolism pathways in strain 1307. The presence and absence (marked with question marks) of genes were predicted based on the annotations from KEGG. Created via BioRender.com. H_2ase, hydrogenase; Nap, periplasmic nitrate reductase; Nrf, nitrite reductase; Nos, nitrous oxide reductase; Fcc, Flavocytochrome c sulfide dehydrogenase; Ttr, tetrathionate reductase; Sqr, sulfide-quinone oxidoreductase; Sox, sulfur-oxidation multienzyme; Psr, thiosulfate reductase; PFK, phosphofructokinase; FBP, Fructose 1,6-bisphosphate; pps, phosphoenolpyruvate synthase; PK, Pyruvate kinase. The pathway map was modified based on the strain genome of *S.* sp. 1612 (Wang et al. 2024).

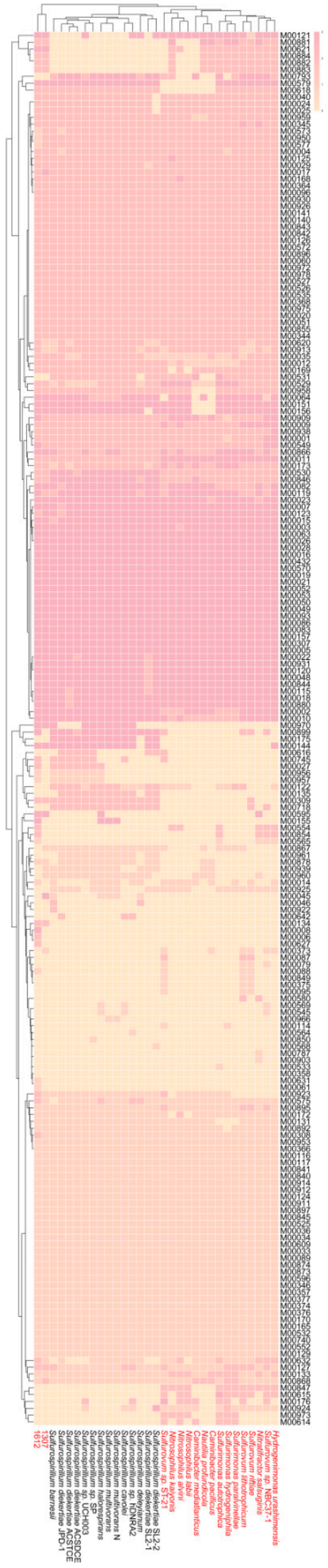

Figure S6 Comparison of KEGG modules between HTV and non-HTV strains.

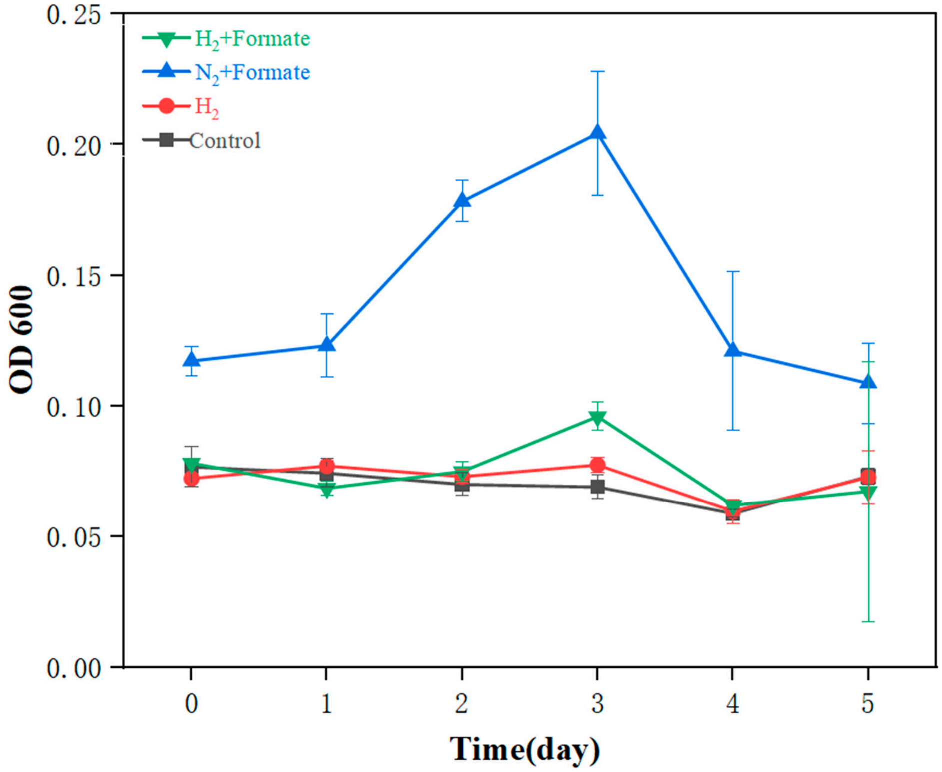

Figure S7 The growth curves for the Electron donor testing. Electron donor utilization was tested by supplementing hydrogen, formate, or a combination of hydrogen and formate, while elemental sulfur (S^0) served as the sole electron acceptor. "Control" refers to the negative control without bacterial inoculum. Cultures were grown in MJ synthetic seawater medium supplemented with 0.1% (w/v) $NaHCO_3$, with S^0 (0.3%, w/v) added as the basal electron acceptor. Formate was supplied at a final concentration of 0.1% (w/v). For experiments without hydrogen, the headspace gas consisted of N_2 (200 kPa). For hydrogen-amended treatments, the headspace gas composition was $N_2/H_2/CO_2$ (50:40:10, 200 kPa).
